# Novel Therapeutic Advances in β-Thalassemia

**DOI:** 10.3390/biology10060546

**Published:** 2021-06-18

**Authors:** Alexandros Makis, Ersi Voskaridou, Ioannis Papassotiriou, Eleftheria Hatzimichael

**Affiliations:** 1 Department of Pediatrics, Faculty of Medicine, University of Ioannina, Stavros Niarchos Avenue, 45110 Ioannina, Greece; 2 Centre of Excellence in Rare Haematological Diseases-Haemoglobinopathies, “Laikon” General Hospital, 11527 Athens, Greece; ersi.voskaridou@gmail.com; 3 Department of Clinical Biochemistry, “Aghia Sophia” Children’s Hospital, 11527 Athens, Greece; ipapassotiriou@gmail.com; 4 Department of Hematology, Faculty of Medicine, University of Ioannina, Stavros Niarchos Avenue, 45110 Ioannina, Greece; ehatzim@uoi.gr

**Keywords:** thalassemia, clinical trial, ineffective erythropoiesis, iron metabolism, gene therapy

## Abstract

**Simple Summary:**

Beta-thalassemia (β-thalassemia) is an autosomal recessive inherited disorder that causes decreased production of hemoglobin. Ineffective erythropoiesis and excess iron deposition are the most significant pathophysiological problems. Chronic red blood cell transfusions along with control of iron overload are the main principles of treatment. Yet, the patients have a problematic quality of life. Recently, novel therapies have emerged based on better knowledge of the pathophysiology of the disease. Aiming at ineffective erythropoiesis through the TGF-β ligand traps, such as luspatercept, has been shown to reduce the transfusion burden. Therapeutic approaches aiming at the iron metabolism mechanisms as well as the pathway of the production of erythroid cyclic guanosine monophosphate are being used in clinical trials with encouraging results. Significant improvements in the technique of hemopoietic stem cell transplantation have been accomplished, with a focus on the improvement of the conditioning regimen and the donor selection. Gene therapy has exhibited remarkable advances using lentiviral β-globin gene insertion techniques or gene editing platforms that target the suppression of γ-globin repressors. All these approaches will have a positive result in the quality of life of thalassemia patients.

**Abstract:**

The main characteristic of the pathophysiology of β-thalassemia is reduced β-globin chain production. The inevitable imbalance in the α/β-globin ratio and α-globin accumulation lead to oxidative stress in the erythroid lineage, apoptosis, and ineffective erythropoiesis. The result is compensatory hematopoietic expansion and impaired hepcidin production that causes increased intestinal iron absorption and progressive iron overload. Chronic hemolysis and red blood cell transfusions also contribute to iron tissue deposition. A better understanding of the underlying mechanisms led to the detection of new curative or “disease-modifying” therapeutic options. Substantial evolvement has been made in allogeneic hematopoietic stem cell transplantation with current clinical trials investigating new condition regimens as well as different donors and stem cell source options. Gene therapy has also moved forward, and phase 2 clinical trials with the use of β-globin insertion techniques have recently been successfully completed leading to approval for use in transfusion-dependent patients. Genetic and epigenetic manipulation of the γ- or β-globin gene have entered the clinical trial setting. Agents such as TGF-β ligand traps and pyruvate kinase activators, which reduce the ineffective erythropoiesis, have been tested in clinical trials with favorable results. One TGF-β ligand trap, luspatercept, has been approved for use in adults with transfusion-dependent β-thalassemia. The induction of HbF with the phosphodiesterase 9 inhibitor IMR-687, which increase cyclic guanosine monophosphate, is currently being tested. Another therapeutic approach is to target the dysregulation of iron homeostasis, using, for example, hepcidin agonists (inhibitors of TMPRSS6 and minihepcidins) or ferroportin inhibitors (VIT-2763). This review provides an update on the novel therapeutic options that are presently in development at the clinical level in β-thalassemia.

## 1. Introduction

Beta-thalassemia (β-thalassemia) is an autosomal recessive inherited disease characterized by decreased production of the β-globin chains of hemoglobin (Hb) A. The normal structure of HbA is two α- and two β-globin chains. Individuals with β-thalassemia are either homozygous or double heterozygotes for mutations in the β-globin gene. The severity of the disease depends on the type of mutation in the β-globin gene and the extent of impairment of β-globin chain production. The β^+^ allele correlates with decreased but not absent production, and the β^0^ with no production. β-thalassemia can be classified as transfusion-dependent thalassemia (TDT) and non-transfusion-dependent thalassemia (NTDT) according to the severity of anemia and the need for transfusions. The heterozygous β-thalassemia trait is a clinically asymptomatic state with a mild microcytic anemia [1].

The mutation in the β-globin gene on chromosome 11 is responsible for the impaired β-globin production and subsequently for the accumulation of unmatched a-globin precipitates. These iron-containing insoluble bodies induce the generation of reactive oxygen species that are deleterious to the cell membrane structures of erythroid cells. The oxidative stress leads to the premature apoptosis of the erythroblasts, which is called ineffective erythropoiesis [2,3]. The changes in membrane proteins of mature red blood cells (RBC), especially the increased expression of phosphatidylserine, lead to hemolysis from the macrophages of the reticuloendothelial system. Furthermore, the overactivation of transforming growth factor-β (TGF-β), which acts as an inhibitor of the final stage of erythropoiesis, has an important role in the process of ineffective erythropoiesis [4].

Ineffective erythropoiesis and peripheral hemolysis lead to severe anemia, tissue hypoxia, and a reactive production of erythropoietin (EPO) with a consequent compensatory increase of the number of bone marrow erythroblasts and extramedullary hematopoiesis with characteristic hepatosplenomegaly. The activated erythroblasts release several proteins, such as erythroferrone (ERFE), which inhibit hepcidin, the master regulator of iron homeostasis. The consequence of low hepcidin levels is the increased intestinal absorption of iron, which, along with chronic hemolysis and RBC transfusions, leads to progressive tissue iron deposition. Excessive free iron has a negative impact in erythropoiesis, creating a vicious cycle between ineffective erythropoiesis and increased iron absorption [5,6,7,8,9,10].

The management of β-thalassemia is optimized to each patient’s clinical course and profile and is primarily focused on the improvement of anemia and the regulation of iron overload and its complications. Endocrinological abnormalities (e.g., hypogonadism, hypothyroidism, insulin resistance/diabetes, growth impairment) are common and are attributable mainly to iron toxicity [11,12]. TDT patients require lifelong RBC transfusions, whereas NTDT patients are transfused upon certain indications, such as sudden Hb decrease, growth delay in children, splenomegaly, bone deformities, extramedullary hematopoiesis, and pulmonary hypertension. In cases of hypersplenism, splenectomy can be performed. Iron chelators, such as desferrioxamine, deferiprone, and deferasirox, are used for the management of excess iron deposition. In the case of severe iron deposition, the iron chelators can be used in combination [13,14,15]. The quality of life of thalassemic patients is affected due to the life-long need for chronic transfusions and iron chelation treatment and the relevant complications. Allogeneic hematopoietic stem cell transplantation (allo-HSCT) is a potential curative treatment for transfusion-dependent patients without iron-related complications, especially at a young age [16].

Recently, novel therapies have emerged which are based on the knowledge of the pathophysiological mechanisms of β-thalassemia. In order to correct the imbalance between the α- and non-α-chains of Hb, agents which promote the production of γ-chains, such as hydroxycarbamide, 5-azacytidine, short-chain fatty acids, and thalidomide, have been used in thalassemia patients and presented partial responses without long-term favorable outcomes [13,17,18]. Furthermore, the stimulation of erythropoiesis has been attempted with the use of EPO with variable results [19].

Further knowledge of the underlying pathways of ineffective erythropoiesis and hemosiderosis has resulted in the emergence of promising novel therapeutic agents, such as TGF-β ligand traps (i.e., luspatercept). Additionally, improvement of HSCT protocols concerning the conditioning regimen, the selection of the donor, and the source of the stem cells is currently under evaluation in clinical trials. Gene therapy with β-globin addition has been shown to be effective and safe in clinical trials and new experimental gene manipulation techniques, such as genome editing, have recently been developed and the first results from clinical trials are very encouraging. The aim of this review is to present the advances in the treatment of β-thalassemia by providing an update on the established and emerging curative and “disease-modifying” (non-curative) treatments (Table 1).

## 2. Curative Treatments

### 2.1. Allogeneic Hematopoietic Stem Cell Transplantation (Allo-HSCT)

Allo-HSCT is a potentially curative treatment for the TDT disease; however, the consequent toxicities and mortality are serious concerns. In TDT patients, the presence of good risk characteristics, according to the Pesaro criteria, is correlated to a successful outcome greater than 90%. The Pesaro criteria are applicable to patients below the age of 16 and include three variables related to iron toxicity: regularity of iron chelation, the presence of hepatomegaly, and the presence of liver fibrosis. The ideal candidates are mainly young children with no comorbidities and a human leukocyte antigen (HLA)-identical sibling donor. The standard myeloablative condition regimens are mainly based on chemotherapy alone, with the use of the alkylating agents busulfan and cyclophosphamide [20,21]. Allo-HSCT in thalassemic patients with high-risk criteria poses difficulties due to elevated rates of graft rejection and transplant-related mortality [16]. Adults with TDT will always be at high risk, but several new conditioning regimens are being evaluated in clinical trials as an effort to improve the transplant outcomes (NCT01050855, NCT00920972, NCT02435901). Favorable results have been reported using modified or reduced intensity conditioning (e.g., treosulfan/thiotepa/fludarabine) or even nonmyeloablative regimens. With these modern promising approaches, both age and Pesaro classification lose much of their predictive value [22,23,24].

The preferable source of stem cells is the bone marrow rather than peripheral blood, possibly due to lower risk of development of chronic graft-versus-host disease. Peripheral blood stem cells have also been used in an effort to decrease the possibility of graft rejection in high-risk thalassemic patients (NCT02105766). Optimally, fully matched sibling donors are preferred, but matched unrelated donors might also be considered (NCT01049854), as well as related cord blood transplants in patients with low-risk criteria. Unrelated umbilical cord blood cells and haploidentical transplants should ideally be performed in clinical trial setting (NCT02126046, NCT00977691, NCT00408447, NCT02504619).

### 2.2. Gene Therapy

**Figure 1:**
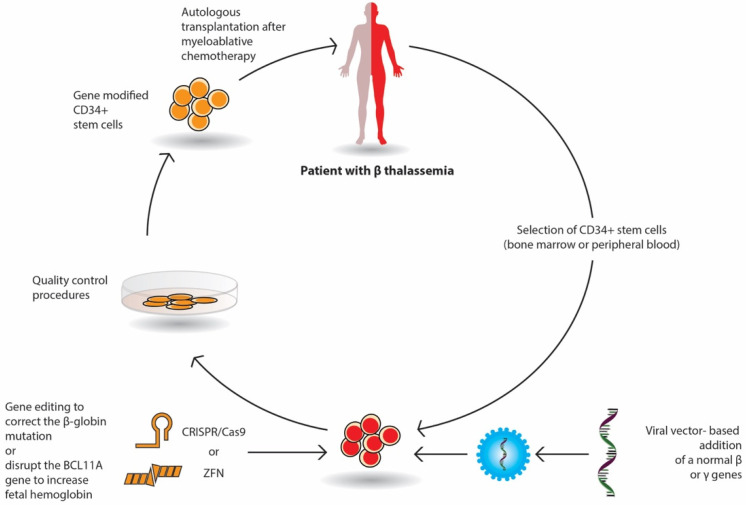
Stepwise procedure of gene therapy by gene addition and by gene editing in β-thalassemia.

Gene therapy by autologous transplantation of genetically manipulated hematopoietic stem cells is a very promising curative option in β-thalassemia. Throughout the last decade, several gene transfer protocols have been comprehensively explored. Among them, lentivirus vectors have been used in thalassemic mouse models and in erythroid stem cells from thalassemic patients. These studies have focused on β or γ-globin addition; the increased expression of γ-globin-activating transcription factors; the silencing of DNA- or RNA-binding proteins that inhibit the expression of γ-globin repressors, such as BCL11A; and the genome editing of β-globin mutations or γ-globin repressors (Figure 1). Currently, several ongoing clinical trials have demonstrated promising results.

The selected CD34+ erythroid progenitor cells (bone marrow or peripheral cells) of the patient are genetically modified either by the viral vector-based addition of a normal β or γ gene or by gene editing with nucleases (Crisp/Cas9 or ZFN), which repair the β globin mutation or delete genomic regions of the BCL11A gene in order to reactivate fetal Hb (HbF) production. The genetically corrected CD34+ stem cells are prepared for autologous transplantation after strict quality control procedures. The patient receives proper myeloablative chemotherapy and then the stem cells are infused in the patient.

#### 2.2.1. Gene Addition

In gene addition, a retroviral or lentiviral vector that encloses the regulatory elements and the β- or γ-globin gene area is inserted into previously collected autologous CD34+ erythroid progenitor cells ex vivo and then infused to the patient who has received a myeloablative busulfan conditioning. Following engraftment, it is expected that β-globin or γ-globin production will be restored, and the α/β imbalance will be reduced (Figure 1).

The outcomes of two phase 1/2 clinical trials (HGB-204/NCT01745120 and HGB-205/NCT02151526) using the LentiGlobin BB305 vector in 22 TDT patients (12–35 years of age) were published in 2018 [25]. The LentiGlobin BB305 vector inserts a functioning version of the *HBB* gene into the patient’s CD34+ erythroid progenitor cells, which encodes HbA with a new T87Q amino acid substitution (HbAT87Q). All patients tolerated the conditioning busulfan regimen with no serious toxicity, and no significant safety concerns regarding the infusion have been reported. Furthermore, regarding the safety of the viral vector, no oncogenic clonal dominance was noted. Hematopoietic engraftment was successful in all patients. Regarding the non–β^0^/β^0^ genotype, all but 1 of the 13 patients became transfusion independent after a median period of 2 years (range, 15–42 months) after the procedure. The Hb levels were between 8.2 and 13.7 g/dL, and the new hemoglobin HbAT87Q varied from 3.4 to 10.0 g/dL. An increase of Hb by approximately 5 g/dL was enough for the HbE/β-thalassemia or β^0^/β^+^ patients to become transfusion independent. As expected, the results were different in patients with the most severe genotype (β^0^/β^0^ or two copies of the IVS1-110 mutation), where transfusion independence requires significantly higher levels of Hb production. In these patients the median transfusion need per year was declined by two thirds, and three patients became transfusion independent.

In order to address this problem, especially in β^0^/β^0^ patients, the sponsor of the trial, bluebird bio, has significantly improved the protocol with the addition of small molecules to the transduction process. This change has a positive effect on the vector copy number in the ongoing phase 3 trials (HGB 207 and HGB-212). A rapid increase of HbAT87Q levels was noticed in most (18/20, 90%) non-β^0^/β^0^ patients, leading to transfusion independence with Hb levels > 9 g/d [26]. Based on these positive outcomes and satisfactory safety reports, the lentiglobin gene therapy (Zynteglo) received conditional EMA in June 2019 for TDT patients ≥ 12 years of age with a non-β^0^/β^0^ genotype who are eligible for stem cell transplantation but do not have a matching related donor.

The primary results of the HGB 212 phase 3 trial from 11 patients with either a β^0^ or IVS-I-110 mutation at both alleles of the *HBB* gene showed that three of four patients stopped transfusions for ≥ 6 months with Hb levels of 10.5–13.6 g/dL at the last visit [27]. The HbAT87Q levels stabilized at a range of 9.5 to 12.6 g/dL 6 months after infusion. After 2 years of follow-up in these four studies (HGB-204, HGB-205, HGB 207, and HGB-212), a long-term follow-up study (LTF-303, NCT02633943) was initiated. The interim results of 32 patients have shown that 64% of the patients from the HGB-204/HGB-205 studies and 90% of the patients of the HGB 207/HGB-212 studies achieved durable and stable transfusion independency [28]. Interim results in pediatric patients in the HGB-207 and HGB-212 showed that children achieved transfusion independence with comparable rates and safety as in adults [29].

Another lentiviral gene-insertion phase 1/2 trial (NCT02453477, SR-TIGET) was recently published [30]. Nine patients (three adults, six children) with β^0^ or severe β^+^ mutations received, with intrabone administration, transduced GLOBE lentiviral vector CD34+ cells. Satisfactory hematopoietic engraftment was achieved in all the patients, with no clonal dominance. Three children stopped transfusions, and the adults also exhibited a significant decrease in transfusion requirements.

A different gene insertion approach has been developed at Boston Children’s Hospital. The target is the *Bcl11a* gene with the use of RNA interference technology. A short hairpin RNA sequence that represses the *Bcl11a* gene is transduced into CD34+ erythroid progenitor cells using a lentivirus. A relevant clinical trial has been started for sickle cell disease patients [31].

#### 2.2.2. Gene Editing

The *Bcl11a* gene located on chromosome 2 is a promising target for gene editing techniques. Two companies, Sangamo Therapeutics/Sanofi, Inc. and Crispr/Vertex, Inc., are studying the use of ZFN (NCT03432364) and CRISPR-Cas9 (NCT03655678) platforms, respectively, in TDT patients. The main idea is to produce minor deletions in the erythroid-specific enhancer region of the *Bcl11a* gene. The major advantage of this approach is that the *HBB* gene is not affected, allowing for the unceasing production of the γ-globin chains. These clinical trials are presently enlisting individuals with thalassemia. Sangamo Therapeutics/Sanofi, Inc. presented preliminary results at the 2019 ASH meeting (NCT03432364) from two patients. Both patients had rapid hematopoietic engraftments with elevated HbF levels [32]. The results of five patients treated with CRISPR-Cas9 gene editing (CTX001 product) were presented at the 2020 ASH meeting. The follow-up was at least 3 months post infusion as of July 2020. The neutrophil and platelet engraftment occurred at the end of the first month, and all patients stopped receiving transfusions after the second month. The first patient enrolled in the study has remained without the need for transfusion for over 15 months. The safety report after CTX001 treatment was similar to that with busulfan myeloablation [33,34].

## 3. “Disease-Modifying” (Non-Curative) Treatments

### 3.1. The TGF-β (Transforming Growth Factor-β) Superfamily Ligand Traps

**Figure 2:**
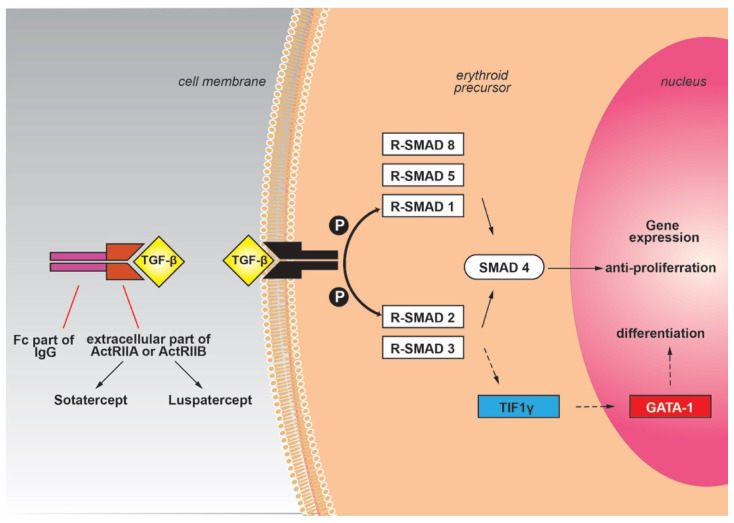
TGF-β signaling pathway and its ligand traps.

The TGF-β signaling is significant for the regulation of essential cellular pathways, especially in the bone and hematopoietic tissue [35], and comprises four similar protein groups: activins, TGF-β, GDFs (growth and differentiating factors), and BMP (bone morphogenetic proteins). The effect of these proteins on the erythroid lineage can be either inductive (TGF-β, BMP4) [36] or inhibitory (activin, GDFs) [37,38]. Their receptors are serine/threonine kinases that stimulate intracellular paths by recruiting the Sma and Mad related proteins (Smad). As a consequence, the Smad proteins (Smad2/3 or Smad1/5/8) in the cytoplasm are phosphorylated and create an oligomeric combination with Smad4 (co-Smad) and insert the nucleus to modify gene transcription. Activins, GDF8, and GDF11 act through the Smad2/3 pathway, whereas BMPs and other GDFs act through the Smad1/5/8 pathway [39]. The Smad2/3 pathway also has the ability to bypass the Smad4 and to alternatively bind to TIF1γ (transcription intermediary factor 1γ), according to the stage of cellular differentiation. TIF1γ stimulates the expression of the key erythroid transcription factor GATA-1 and promotes the differentiation of erythroid lineage [40] (Figure 2).

Therefore, the Smad2/3 signaling has appeared as a significant regulatory mechanism of erythropoiesis with a balancing activity according to the implicated pathway. The Smad2/3-Smad4 pathway inhibits the proliferation of the erythrocyte progenitor cells, while the Smad2/3-TIF1γ promotes the differentiation. It seems that this orchestrated signaling is necessary for the regular differentiation and cessation of erythropoiesis. The overactivation or dysregulation of Smad2/3-Smad4 signaling has been implicated in illnesses with anemia caused by ineffective erythropoiesis, such as myelodysplastic syndromes or β-thalassemia [4]. Therefore, inhibitors of the TGF-β family that sequester the Smad2/3-Smad4 signaling could promote the end phase of erythropoiesis with a beneficial result in these diseases.

Altered receptors of activin (activin receptor-II trap ligands) have been derived from the colligation of the extracellular part of the activin receptor (ActRIIA or ActRIIB) with the Fc part of IgG immunoglobulin. The presence of the Fc domain not only stabilizes the fusion trap but also increases the half-life in the circulation through its interplay with the FcRn (neonatal Fc-receptor) recycling mechanism. This interaction is beneficial because it prolongs the effect of the drug [41]. In this form, the Fc-IgG fusion displays an increased binding tendency to activins or other TGF-β proteins and reduces the stimulation of the Smad pathway. ACE-011 (sotatercept) is an Fc-IgG fusion trap that derives from the combination of the extracellular fragment of ActRIIA with the Fc part of human IgG. ACE-536 (luspatercept) is a fusion of an altered extracellular domain of ActRIIB with the Fc region of human IgG1, while RAP-536 involves mouse IgG2a [42] (Figure 2). RAP-536 fusion proteins have been used in mouse models with thalassemia, with beneficial effects on the reduction of ineffective erythropoiesis, splenomegaly, and iron overload. The use of ACE-536 (luspatercept) reduced the activation of the Smad2/3-Smad4 pathway and promoted the differentiation of the erythroid lineage by reestablishing GATA-1 function, likely by favoring the Smad2/3-TIF1γ pathway over Smad2/3-Smad4. Furthermore, upregulation of *HSP70* led to the amelioration of oxidative stress and the promotion of erythroid differentiation [43,44,45,46]. The exact mechanism of action of luspatercept is not fully defined, and further research is needed to reveal the cellular consequences of TGF-β inhibition.

The use of luspatercept in adult thalassemia subjects has been initially evaluated in a phase 2 trial (NCT01749540). In this study, the subcutaneous use of luspatercept improved Hb levels and reduced transfusion requirements [47]. In particular, luspatercept led to a mean increase in Hb of ≥ 1.5 g/dL for at least 2 weeks in more than half (58%) of the NTDT patients, while in 81% of TDT patients a ≥ 20% decrease in RBC transfusion requirements was recorded. The most common grade 1 to 2 adverse events were headache, bone pain, and myalgia [47]. This 24-week dose-finding study was followed by a 5-year extension phase, presently continuing (NCT02268409). Comparable results were obtained with sotatercept; however the drug was not selected for further phase 3 studies, mainly because, distinct to luspatercept, it is a less specific activin receptor-II ligand trap, may be less efficacious in treating anemia and have more off-target effects [48].

Regarding luspatercept, the encouraging results from the phase 2 trial led to the phase 3 BELIEVE trial (NCT02604433), which was recently completed. The trial was designed to define the efficacy and safety of luspatercept in TDT adults [49], who were randomized to obtain subcutaneously either luspatercept at an initial dose of 1.0 mg/kg with a gradual increase up to 1.25 mg/kg or a placebo every 3 weeks for ≥ 48 weeks. The primary endpoint was a ≥ 33% reduction in transfusions, with a reduction of ≥ 2 RBC units during weeks 13 to 24. The secondary endpoints comprised a ≥ 33% reduction in transfusion load and at least 2 RBC units at weeks 37 to 48, and a ≥ 50% reduction in transfusions and at least 2 RBC units at weeks 13 to 24. The median age of the patients was 30 years (range 18–66 years), and 58% were women. The β^0^/β^0^ genotype was observed in 30% patients in the luspatercept group and 31% patients in the placebo group. The primary endpoint was significantly achieved in the luspatercept group: 21.4% vs. 4.5% achieved a reduction of at least 33% in the transfusions during weeks 13 to 24 [49].

All the subgroups (β^0^/β^0^, β^0^/β^+^, β^+^/β^+^, HbE/β-thalassemia) benefited consistently from luspatercept treatment, even the difficult-to-treat patients, such as those with the β^0^/β^0^ genotype (although the magnitude of benefit was lower) or patients receiving more than 6 RBC units/12 weeks at baseline. While response rates were lower in patients with the most severe disease (β^0^/β^0^), clinically significant declines in transfusion load were observed across genotypes. A ≥ 33% decrease in the transfusions at weeks 37–48 was accomplished by 19% of the luspatercept group compared to 3.6% of the placebo group. Regarding the additional target of achieving a ≥ 33% decrease in transfusions throughout any successive 12 weeks of the trial, 70.5% of the luspatercept group reached this level compared to 29% of the placebo group, while for any 24-week period, the difference was 41.1% and 2.7%, respectively. Among patients who achieved a decline of at least 33% during any 12 weeks, 80.4% had at least two episodes of response, and 51.3% had at least four episodes of response. Treatment-emergent adverse effects were similar with data previously described in the phase 2 study. Adverse effects in the luspatercept arm included bone pain (19.7%), arthralgia (19.3%), dizziness (11.2%), hypertension (8.1%), and hyperuricemia (7.2%). Bone pain was more frequent during the first 12 weeks in both arms and could be alleviated with regular analgesia. Thromboembolic events occurred in eight patients (3.6%) in the luspatercept arm and in one patient (0.9%) in the placebo arm. All eight patients in the luspatercept group had undergone splenectomy and had at least one risk factor for thromboembolic events. The discontinuation rate due to an adverse event was 5.4% for the luspatercept arm and 0.9% for the placebo, and no deaths were reported in either treatment groups [49]. As expected, recent data on long-term follow up have shown that the addition of luspatercept in standard care had sustained long-term efficacy [50], reduced the risk of iron overload complications [51], and improved the health-related quality of life [52].

In order to study the safety and pharmacokinetics in TDT children, a phase 2 clinical trial (NCT04143724) has been planned but is not yet recruiting patients. A major concern in children is the potential toxicity of luspatercept in the developing hematopoietic system and other vital organs. Furthermore, interfering with the BMP pathway may compromise growth in an immature skeletal system.

Luspatercept could also have a place in the treatment of NTDT patients. The BEYOND trial is a phase 2 study to define the effectiveness and safety of luspatercept in adults with NTDT (NCT03342404) [53]. The primary objective is the increase of mean Hb without any transfusions over a 12-week period, from week 13 to 24, compared to the initial phase. The study was completed in September 2020, and the results are expected.

In summary, the subcutaneous use of luspatercept every 3 weeks demonstrated significant reductions in RBC transfusion burden in TDT adult patients and was granted FDA (Food and Drug Administration) and EMA (European Medicines Agency) approval for TDT patients. It seems that luspatercept will have a significant place in the real-life management of TDT patients.

### 3.2. Pyruvate Kinase Activation

Pyruvate kinase (PK) is the enzyme that plays a significant role in the last stage of glycolysis in the RBC, the conversion of phosphoenolpyruvate to pyruvate in order to generate ATP. Therefore, PK is necessary for the energy production in the RBC. In patients with PK deficiency, an autosomal recessive disease, the energy-depleted erythrocytes are prone to hemolysis. Additionally, the ATP-deficiency in the erythroblasts leads to ineffective erythropoiesis. Patients with PK deficiency have symptoms and signs due to ineffective erythropoiesis and chronic hemolysis [54] and the clinical picture resembles NTDT. The oral use of the PK activator AG-348 (mitapivat) in healthy subjects has been shown to increase ATP [55] and has been proven to be efficient and safe in PK deficiency patients [56].

The promising results of mitapivat in PK deficiency, a disease resembling NTDT, led to the use of mitapivat in NTDT. The results showed a significant decrease of reactive oxygen species levels and an improvement of ineffective erythropoiesis. Moreover, a reduction of liver iron deposition and an increase of hepcidin levels were observed [57]. The interim results from a phase 2 trial (NCT03692052) of mitapivat in 13 adults with NTDT (α- or β-thalassemia) were presented at the 2020 ASH annual meeting. Mitapivat was given orally twice daily for 6 weeks, with a starting dose of 50 mg and a possibility to increase to 100 mg at the 6th week. The majority of the patients (92.3%, 4/4 with α-thalassemia, 8/9 with β-thalassemia) achieved an Hb increase from baseline above 1.0 g/dL after a median of 3.1 weeks (range 1.4–7.1). During the period of 4–12 weeks, the mean Hb increase from baseline was 1.34 g/dL with concomitant amelioration of hemolysis indices. No serious adverse events were reported [58]. Phase 3 studies will assess the efficacy and safety of mitapivat in TDT and NTDT patients (α- or β-thalassemia) (NCT04770779, NCT04770753).

### 3.3. Phosphodiesterase 9 Inhibition

The alteration of intracellular cyclic guanosine monophosphate (cGMP) is a novel therapeutic objective for sickle cell disease and thalassemia. The cGMP-dependent pathway is significant for the production of HbF and has multiple roles in vascular biology. As phosphodiesterase (PDE) 9 selectively degrades cGMP in erythropoietic cells, the use of inhibitors of PDE9 can result in increased cGMP levels and the reactivation of HbF [59,60].

IMR-687 is a novel agent that has been developed for the inhibition of PDE 9. The oral use of IMR-687 in sickle cell disease patients has been recently completed (NCT03401112) and has shown to stimulate HbF production and to improve Hb levels and hemolysis indices [61]. A similar phase 2 study has been launched in order to evaluate the safety and tolerability of IMR-687 given once daily for 36 weeks in TDT and NTDT adult thalassemic patients (NCT04411082).

### 3.4. Iron Metabolism Manipulation

Hepcidin, a protein produced from the hepatocytes, is the key controller of iron metabolism. Hepcidin degrades ferroportin, the main iron exporter in intestinal cells; macrophages of the reticuloendothelial system; and the hepatocytes. In β-thalassemia, hepcidin production is inhibited through the action of ERFE, contributing to increased intestinal iron absorption and tissue deposition [7]. Restoration of hepcidin levels could improve iron overload and ineffective erythropoiesis [62].

Hepcidin production is mainly regulated by matriptase-2 (MT-2), a transmembrane serine protease, which is encoded by the *TMPRSS6* gene. MT-2 inhibits hepcidin activation by cleaving membrane hemojuvelin [63]. In mouse models with NTDT, the inhibition of *TMPRSS6* with silencing RNAs or antisense oligonucleotides caused a rise of hepcidin and an amelioration of anemia and iron deposition [64,65,66,67]. Phase 2 clinical trials are already recruiting NTDT patients in order to assess the efficacy, safety, and pharmacokinetics of the short interfering RNAs, SLN124 (NCT04718844), and IONIS TMPRSS6-LRx (NCT04059406).

The use of minihepcidins, which act as hepcidin agonists, has been proven to improve ineffective erythropoiesis and splenomegaly in a TDT mouse model [68]. Recent initial results of a phase 2 trial (NCT04054921) on the subcutaneous use of PTG-300, a hepcidin mimetic analog, have shown a reduction in serum iron parameters and a 20% or greater reduction in transfusions, with mild adverse effects [69]. Another approach is to restrict the availability of iron, targeting both the iron overload and the ineffective erythropoiesis, with the use of ferroportin inhibitors (VIT-2763). These agents could be beneficial in NTDT. In an NTDT mouse model, the oral use of VIT-2763 along with deferasirox reduced liver iron and improved anemia [70]. A phase 1 study in healthy volunteers has shown that the drug was well accepted with no significant safety concerns along with a transient decrease in mean serum iron levels and transferrin saturation [71]. A phase 2 study of VIT-2763 in NTDT (VITHAL, NCT04364269) is already recruiting patients.

## 4. Conclusions—Key Points

In β-thalassemia, the globin chain inequality, ineffective erythropoiesis, and iron tissue deposition are the main underlying pathophysiological mechanisms that lead to the complications of the disease. Recent research on these mechanisms has revealed novel curative and “disease-modifying” therapeutic approaches.

Clinically meaningful progress in the techniques of allo-HSCT has been accomplished mainly in the area of the conditioning regimen and donor selection. Gene manipulation studies have exhibited remarkable advances. The lentiglobin gene therapy (Zynteglo) has received conditional EMA approval for TDT patients with non-β^0^/β^0^ genotype who are ≥12 years of age and who are eligible for allo-HSCT but do not have a suitable donor. The need for a specialized center for the procedure as well as the high financial cost are barriers to the widespread application of gene therapy. Furthermore, long-term safety data are needed regarding toxicity due to the conditioning regimen and, potentially, to insertional mutagenesis. However, gene therapy has more a measurable and objective effect than luspatercept and may lead to transfusion independence.

A very recent and highly sophisticated approach is the application of epigenetic and genomic editing methods that target the silencing of γ-globin repressors, such as BCL11A, or the correction of β-globin gene. Remarkable results have been reported in preclinical studies, while the preliminary results from clinical trials are more than promising.

A phase 3 trial in TDT patients has shown that the subcutaneous administration of luspatercept, a TGF-β family member inhibitor, led to a substantial decrease in transfusions, with a favorable safety profile. These findings led to its approval by the FDA and the EMA for treatment of anemia in adult TDT patients. It seems that luspatercept will have a significant place in the treatment algorithm of TDT patients, and its real-world application is expected to offer further information. It is a pharmacological therapy that can be an additive to standard care with manageable side effects that allows the patient to discontinue the drug when needed or in cases of poor response, whereas this in not realistic in gene therapy. It can also be an alternative option in cases of young individuals above 18 years of age who do not have the criteria for transplantation or the availability of a matching related donor. The use of luspatercept is also being studied in NTDT patients, and clinical trials have been designed for children. Furthermore, the fact that luspatercept reduces the need for transfusions will be beneficial in health care systems that have a shortage of blood products, especially during this period of the SARS-CoV-2 pandemic.

Another agent that improves ineffective erythropoiesis is mitapivat, a PK activator. The oral administration of mitapivat in a small number of patients has shown promising results in ongoing clinical trials. An alternative way to improve anemia in thalassemic patients is to stimulate HbF production with the use of phosphodiesterase 9 inhibitors, such as IMR-687. This agent has shown encouraging results in sickle cell disease patients and is currently being tested in aduts with TDT and NTDT. A different therapeutic approach is to target the dysregulation of iron homeostasis and indirectly improve erythropoiesis, by using hepcidin agonists (inhibitors of TMPRSS6 and minihepcidins) or ferroportin inhibitors (VIT-2763).

To conclude, all these emerging treatment modalities require long-term experience in order to further establish their efficacy and safety. Another concern is the availability and the high cost, especially in low- and middle-income countries. Should all modalities be available, a patient-centered approach could be pursued. For individuals with NTDT, transfusions are given as needed in certain clinical settings. In TDT, the mainstream treatment is chronic transfusions to alleviate anemia and suppress extramedullary hematopoiesis, with parallel management of the excess free iron and the complications of the disease. The quality of life is affected, and some adult patients may consider the option of disease-modifying agents which improve erythropoiesis, such as luspatercept. If a curative and permanent treatment is considered, allo-HSCT can be offered with a good outcome, especially in children. Matched related transplantation is the preferable approach, but other alternatives can be considered in terms of donor selection and conditioning regimens. In the case of a non-suitable donor, gene therapy with β-gene addition is an option in certified transplant centers.

## Figures and Tables

**Table 1:** Clinical trials of novel treatment in β-thalassemia.

Treatment Modality	Mechanism	Route	Phase	ClinicalTrials.gov (8 May 2021)	Status	Institution/ Developer
Luspatercept	Ligand trap TGF beta superfamily	Subcutaneous	2	NCT01749540	Completed	Acceleron Pharma, Celgene Corporation
			2, extension study 2 2	NCT02268409 NCT03342404 NCT04143724	Completed Open Not yet recruiting	
			3	NCT02604433	Completed	
Mitapivat	Pyruvate kinase activation	Oral	2	NCT03692052	Open	Agios Pharmaceuticals
TMPRSS6-LRx	Matriptase-2 inhibition, hepcidin activation	Subcutaneous	2	NCT04059406	Open	Ionis Pharmaceuticals
SLN124	Matriptase-2 inhibition, hepcidin activation	Subcutaneous	2	NCT04718844	Open	Silence Therapeutics plc
PTG-300	Hepcidin analog	Subcutaneous	2	NCT04054921	Completed	Protagonist Therapeutics, Inc.
VIT-2763	Ferroportin inhibition	Oral	2	NCT04364269	Open	Vifor (International) Inc.
IMR-687	Phosphodiesterase 9 inhibition, HbF stimulation	Oral	2	NCT04411082	Completed	Imara, Inc.
HSCT	Reduced intensity conditioning	Matched sibling donor	1, 2	NCT00920972, NCT01050855, NCT02435901	Open Open Completed	US, Canada US, Canada US, Canada
		Family related matched donor or cord	2	NCT00408447	Active not recruiting	Columbia University, USA
HSCT	Matched unrelated		2	NCT01049854	Completed	New York Medical College, USA
HSCT	Nonmyeloablative haploidentical	Haploidentical transplants	1,2	NCT00977691	Active not recruiting	National Heart, Lung, and Blood Institute (NHLBI)
HSCT	Nonmyeloablative peripheral blood mobilized	Allogeneic peripheral blood stem cell	1,2	NCT02105766	Open	National Heart, Lung, and Blood Institute (NHLBI)
HSCT	Umbilical cord blood	Umbilical cord stem cells	1	NCT02126046	Open	Nanfang Hospital of Southern Medical University
HSCT CordIn™	Umbilical cord blood-derived ex vivo stem cells	Ex vivo umbilical cord stem cells	1	NCT02504619	Completed	Gamida Cell ltd
LentiGlobin BB305 vector	β-globin gene addition	Ex vivo autologous CD34+ stem cell transduction	1	NCT02151526	Completed	bluebird bio, France
			1/2 3	NCT01745120 NCT03207009	Completed Open	bluebird bio, Northstar Study
TNS9.3.55 Lentiviral Vector	β-globin gene addition	Ex vivo autologous CD34+ stem cell transduction	1	NCT01639690	Active not recruiting	Memorial Sloan Kettering Cancer Center, USA
GLOBE Lentiviral Vector	β-globin gene addition	Ex vivo autologous CD34+ stem cell transduction	1	NCT02453477	Active not recruiting	IRCCS San Raffaele, Italy
CTX001	*BCL11A* gene editing	Ex vivo autologous CD34+ stem cell transduction	1/2	NCT03655678	Open	Vertex Pharmaceuticals Incorporated CRISPR Therapeutics
ST-400	*BCL11A* gene editing	Ex vivo autologous CD34+ stem cell transduction	1/2	NCT03432364	Active not recruiting	Sangamo Therapeutics Sanofi

## Data Availability

Not applicable.
